# Low Parathyroid Hormone *Versus* Secondary Hyperparathyroidism and Survival in Patients Undergoing Hemodialysis: A Propensity-Matched Analysis

**DOI:** 10.3389/fendo.2022.869330

**Published:** 2022-05-13

**Authors:** Wang Guo, Huixian Zhang, Yamei Zhang, Hongdong Huang, Wenhu Liu, Zongli Diao

**Affiliations:** Department of Nephrology, Beijing Friendship Hospital, Capital Medical University, Beijing, China

**Keywords:** low parathyroid hormone, secondary hyperparathyroidism, hemodialysis, all-cause death, cardio-cerebrovascular death

## Abstract

**Introduction:**

Low serum parathyroid hormone (PTH) and secondary hyperparathyroidism (SHPT) are very common in patients undergoing hemodialysis. However, it remains unclear which of these has a lower mortality.

**Objective:**

In this study, we compared outcomes between hemodialysis patients with low PTH and those with SHPT.

**Methods:**

This was a multi-center, retrospective, matched cohort study. Median intact PTH (iPTH) was used as the cutoff for allocating participants to low PTH (iPTH<100 pg/mL) and SHPT groups (iPTH ≥600 pg/mL). Sex, diabetes, age, and dialysis vintage were matched between the groups. The primary outcome was all-cause death at 72 months.

**Results:**

The study cohort comprised 2282 patients (1166 in each study group). Prior to matching, the primary outcome occurred in 429/1166 patients (36.79%) in the low PTH group and in 284/1116 (25.45%) in the SHPT group. There were no significant differences in all-cause death between the groups according to multivariable Cox regression (P=0.423). The hazard ratio for low PTH versus SHPT was 1.08 (95% confidence interval, 0.90–1.30). Propensity matching created 619 pairs of patients. Baseline characteristics, including age, sex, diabetes, and dialysis vintage were comparable between the groups. The primary outcome occurred in 195/619 patients (31.50%) in the low PTH group and in 193/619 (31.18%) in the SHPT group. There were no significant differences in all-cause death between the groups according to multivariable Cox regression (P=0.43). The adjusted hazard ratio for low PTH versus SHPT was 1.10 (95% confidence interval, 0.87–1.39).

**Conclusions:**

Hemodialysis patients with low PTH have similar all-cause death rates to the rates for those with SHPT.

## Introduction

Abnormal serum parathyroid hormone (PTH) concentrations are common in patients undergoing maintenance hemodialysis and are associated with severe consequences, including mortality and cardiovascular events (1–3). However, PTH concentrations are controlled within a target range of approximately 100–600 pg/mL in only approximately 50% of patients, with reported rates of low PTH (<100 pg/mL) and secondary hyperparathyroidism (SHPT, >600 pg/mL) of approximately 40% and 10%, respectively (4).

The impact of serum PTH concentration on risk of death in hemodialysis patients is controversial. Most studies suggest that patients with PTH concentrations within the normal range have the lowest mortality (5, 6). However, some studies have reported only a weak association between serum PTH and the risk of death in patients with chronic kidney disease (7, 8). Some have even suggested that patients with low PTH concentrations have the lowest mortality (9).

Both low PTH and SHPT are reportedly independent risk factors for death and cardiovascular disease in hemodialysis patients (10–12). Low PTH is a risk factor for vascular calcification and death (13, 14), possibly because patients with low PTH are likely to have low-turnover bone disease, which may weaken the bone buffering capacity for serum phosphorus and calcium (15).

Total parathyroidectomy is an effective means of managing severe SHPT (16, 17). However, this surgery can easily result in low PTH concentrations, which may be associated with increased mortality. It is still unclear whether patients with SHPT or those with low PTH have worse prognoses. If patients with low PTH have better prognoses than those with SHPT, the possibility of total parathyroidectomy causing low PTH concentrations is acceptable.

In our previous study, hemodialysis patients with low PTH concentrations had a higher mortality rate than the rate for those with SHPT (18). However, when we matched for age, we found no statistically significant difference in mortality between patients with low PTH and those with SHPT. This discrepancy may have been attributable to an imbalance in baseline characteristics, such as age and diabetes, between the two groups. Both age and diabetes are very important risk factors for the prognosis of patients undergoing hemodialysis. Given that age and diabetes are very influential, multivariable analysis may not eliminate their confounding effects.

In this study, we compared the outcomes of a large matched cohort of hemodialysis patients with low PTH or with SHPT.

## Patients and Methods

### Study Design

This was a retrospective, matched, cohort study. We extracted and analyzed data from a 7-year cohort of all maintenance hemodialysis patients from 138 dialysis facilities of the Beijing Hemodialysis Quality Control and Improvement Center, a large dialysis organization in China.

### Study Cohort

Between January and December 2014, all hemodialysis patients registered with the Beijing Hemodialysis Quality Control and Improvement Center were screened for eligibility according to the following inclusion and exclusion criteria:

Inclusion criteria: (1) age: 18–80 years; (2) intact PTH (iPTH) <100 pg/mL or ≥600 pg/mL; (3) iPTH measured at least every 6 months, with the median iPTH used if iPTH was measured more frequently; and (4) regular hemodialysis for ≥6 months.

Exclusion criteria: (1) median serum albumin <30 g/L; (2) median hemoglobin <90 g/L; and (3) malignant tumor.

The patients were divided into low PTH (iPTH <100 pg/mL) and SHPT groups (iPTH ≥600 pg/mL) according to their median iPTH during the study period. Data from eligible patients were collected until death, kidney transplant, transfer to peritoneal dialysis, or December 2019.

### Outcomes

The primary outcome of this study was all-cause death at 72 months, and the secondary outcome was cardio-cerebrovascular death at 72 months. Cardio-cerebrovascular death comprised cardiac arrest and death from heart failure, ischemic heart disease, or cerebrovascular disease.

### Propensity Matching

It was anticipated that the baseline characteristics of the patients in the low PTH and SHPT groups would differ significantly. To avoid confounding as a result of these differences, the following variables were matched between the two study groups: sex, diabetes, age ( ± 2 years), and dialysis vintage ( ± 12 months).

We also performed a subgroup analysis by dividing the low PTH group into three subgroups in accordance with the median iPTH: low PTH Group 1 (iPTH 60–100 pg/mL), low PTH Group 2 (iPTH 30–60 pg/mL), and low PTH Group 3 (iPTH <30 pg/mL). These three subgroups were also matched with the SHPT group, using the variables listed above.

In addition, we compared the prognosis between three groups by analyzing matched study cohorts, as follows: low PTH group (iPTH <100 pg/mL), medium PTH group (iPTH 100–600 pg/mL), and SHPT group (iPTH ≥600 pg/mL). These three subgroups were also matched with each other, using the variables listed above.

### Statistical Analysis

Continuous variables are presented as mean ± standard deviation or median and interquartile range. We tested for differences in the patients’ characteristics using Student’s *t*-test, paired-samples *t*-test, Mann–Whitney U test, Wilcoxon’s test, or the Friedman test, as appropriate. Categorical variables are expressed as frequencies and percentages and were compared using the χ^2^ test or Fisher’s exact test.

Two models were applied: (1) survival curves were estimated by Kaplan–Meier analysis, and the curves were compared by the log-rank test as a univariable model with two or three groups divided by the median iPTH; and (2) multivariable Cox regression with adjustment for categorical variables (low PTH, medium PTH, SHPT, sex, diabetes) and continuous variables (age, dialysis vintage, and median values for phosphorus, calcium, hemoglobin, and albumin concentrations).

We pre-specified three subgroups for analysis as specified under the heading “Propensity matching” above. These three subgroups were each matched to the SHPT group, and the outcomes were compared between each subgroup and the SHPT group.

P <0.05 was considered to denote statistical significance, and all analyses were performed using IBM SPSS 26.0 (IBM Corp., Armonk, NY, USA).

## Results

### Study Cohort

**Figure 1:**
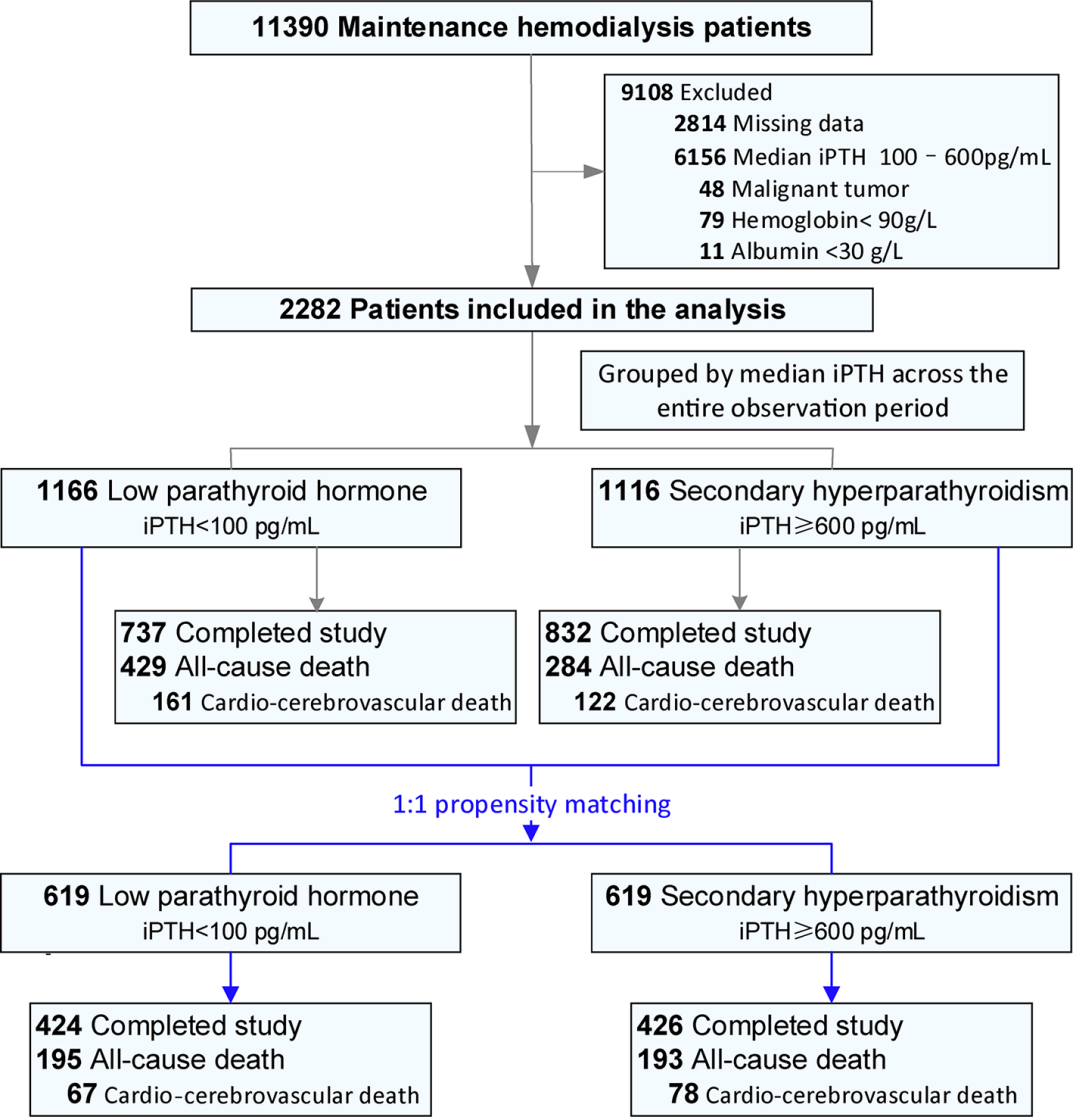
Flow diagram of the study.

**Figure 1** is a flow chart of the study. Between 1 January and 31 December 2014, 11 390 maintenance hemodialysis patients were registered with the Beijing Hemodialysis Quality Control and Improvement Center. Of these, 2282 patients (1166 in the low PTH group and 1116 in the SHPT group) were included in this study. In the unadjusted cohort, the median durations of follow-up were 61.94 (28.79–72.00) months and 72.00 (41.70–72) months in the low PTH and SHPT groups, respectively. Both age and the proportion of patients with diabetes were significantly higher in the low PTH group than the values in the SHPT group. The dialysis vintage was significantly shorter in the low PTH group than that in the SHPT group. Propensity matching resulted in 619 pairs of patients. The two groups were well balanced with respect to age, sex, dialysis vintage, diabetes history, and primary cause of renal failure (**Table 1**).

**Table 1:** Patients’ baseline characteristics in the unadjusted and propensity-matched cohorts.

Characteristic	Unadjusted Cohort				Propensity Matched Cohort		
	Low PTH group (n =1166)	SHPT group (n = 1116)		P value	Low PTH group (n = 619)	SHPT group (n = 619)	P value
Median age (IQR) –year	59.4 (51.5–68.4)	52.0 (42.3–61.3)		<0.001	56.2 (48.8–63.7)	56.5 (48.7–63.9)	0.952
Male sex – no. (%)	600 (51.5)	663 (59.4)		<0.001	309 (49.9)	309 (49.9)	NA
Median dialysis vintage (IQR) –month	27.0 (4.2–68.4)	56.0 (25.5–92.2)		<0.001	49.5 (17.1–85.1)	50.73 (22.03–85.22)	<0.001
Cause of ESRD– no. (%)				<0.001			0.928
Diabetic nephropathy	351 (30.1)	125 (11.2)			99 (16.0)	92 (14.9)	
Glomerulonephritis	321 (27.5)	480 (43.0)			236 (38.1)	235 (38.0)	
Hypertensive nephropathy	143 (12.3)	180 (16.1)			85 (13.7)	102 (16.5)	
Tubulointerstitial nephritis	56 (4.8)	69 (6.2)			37 (6.0)	38 (6.1)	
Others	98(8.4)	108(9.7)			61(9.9)	68(11.00)	
Unknown	197(16.9)	154(13.8)			101(16.3)	84(13.6)	
Diabetes	487 (41.8)	205 (18.4)		<0.001	146 (23.6)	146 (23.6)	NA
iPTH (pg/mL)							
Mean ± SD	56.9 ± 27.9	1001.3 ± 459.2			53.7 ± 28.5	989.1 ± 451.7	
Median (IQR)	60 (33.5–80.8)	835.2 (693.6–1135.9)			55.8 (30.3–79.2)	810.9 (686.4–1131.0)	
Calcium (mmol/L)^*^	2.26 ± 0.21	2.32 ± 0.21		<0.001	2.25 ± 0.22	2.31 ± 0.22	<0.001
Phosphate (mmol/L) ^*^	1.60 ± 0.40	2.04 ± 0.40		<0.001	1.64 ± 0.40	2.00 ± 0.40	<0.001
Albumin (g/dL) ^*^	3.90 ± 0.31	3.98 ± 0.27		<0.001	3.94 ± 0.30	3.89 ± 0.25	<0.001
Hemoglobin (g/dL) ^*^	11.3 ± 0.9	11.3 ± 1.0		0.770	11.3 ± 0.8	11.2 ± 0.9	0.129

^*^Presented as means ± SD.

Normally distributed data are presented as means ± SDs and non-normally distributed data as medians.

Low PTH group (iPTH <100 pg/mL); SHPT group (iPTH ≥600 pg/mL).

ESRD, end-stage renal disease; iPTH, intact parathyroid hormone; IQR, interquartile range; NA, not applicable; Low PTH, low parathyroid hormone; SD, standard deviation; SHPT, secondary hyperparathyroidism.

### Primary Outcome

**Figure 2:**
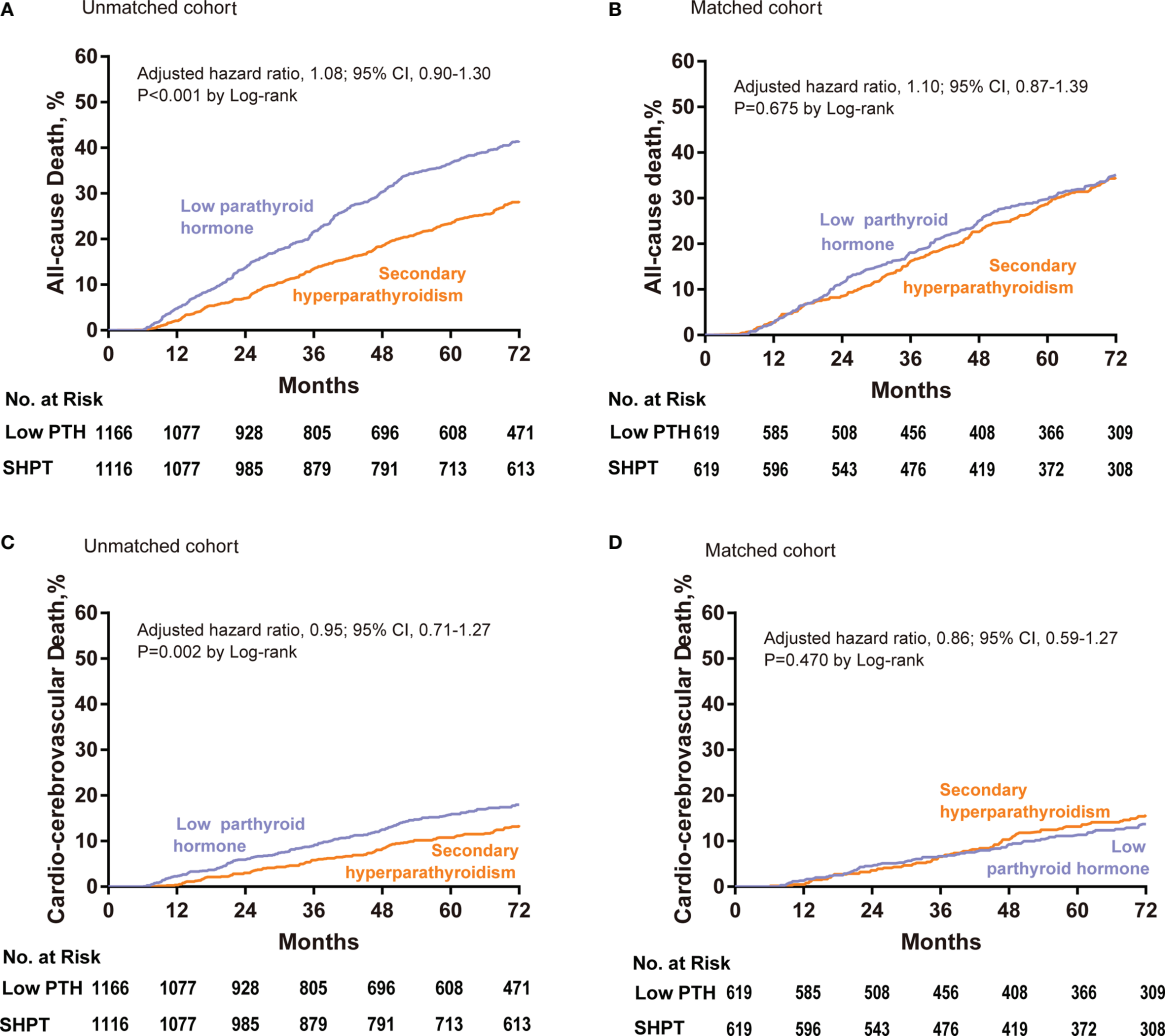
Cumulative event rates at 72 months for all-cause death among patients with low PTH and SHPT in the unadjusted **(A)** and propensity-matched cohorts **(B)**. Cumulative event rates at 72 months for cardio-cerebrovascular death among patients with low PTH and SHPT in the unadjusted **(C)** and propensity-matched cohorts **(D)**. Hazard ratios are based on multivariable Cox regression analyses. PTH, parathyroid hormone; SHPT, secondary hyperparathyroidism.

In the unadjusted cohort, the primary outcome occurred in 429/1166 patients (36.79%) in the low PTH group and in 284/1116 (25.45%) patients in the SHPT group. Kaplan–Meier survival curves according to study group are shown in **Figure 2A** (log-rank test, P<0.001). According to the results of the multivariable Cox regression analysis, there was no significant difference in all-cause death between the low PTH and SHPT groups (P=0.423; hazard ratio (HR) for low PTH versus SHPT, 1.08; 95% confidence interval (CI), 0.90–1.30).

In the propensity-matched cohort, the primary outcome occurred in 195/619 patients (31.50%) in the low PTH group and in 193/619 (31.18%) patients in the SHPT group. Kaplan–Meier survival curves according to study group are shown in **Figure 2B** (log-rank test, P=0.68). Furthermore, according to the results of the multivariable Cox regression analysis, there was no significant difference in all-cause death between the low PTH and SHPT groups (P=0.43; adjusted HR for low PTH versus SHPT, 1.10; 95% CI, 0.87–1.39).

### Secondary Outcome

In the unadjusted cohort, cardio-cerebrovascular death occurred in 161/1166 patients (13.81%) in the low PTH group and in 122/1116 (10.93%) patients in the SHPT group. Kaplan–Meier survival curves according to study group are shown in **Figure 2C** (log-rank test, P=0.002). According to the results of the multivariable Cox regression analysis, there was no significant difference in the rates of cardio-cerebrovascular death between the low PTH and SHPT groups (P=0.718; HR for low PTH versus SHPT, 0.95; 95% CI, 0.71–1.27).

In the propensity-matched cohort, cardio-cerebrovascular death occurred in 67/619 patients (10.82%) in the low PTH group and in 78/619 (12.60%) patients in the SHPT group. Kaplan–Meier survival curves according to study group are shown in **Figure 2D** (log-rank test, P=0.470). According to the results of the multivariable Cox regression analysis, there was no significant difference in the rates of cardio-cerebrovascular death between the low PTH and SHPT groups (P=0.459; HR for low PTH versus SHPT, 0.86; 95% CI, 0.59–1.27).

### Subgroup Analysis for All-Cause and Cardio-Cerebrovascular Death

We divided the patients in the low PTH group into the following three subgroups according to iPTH concentration: low PTH Group 1 (n=584 pairs; 60–100 pg/mL), low PTH Group 2 (n=336 pairs; 30–60 pg/mL), and low PTH Group 3 (n=246 pairs; <30 pg/mL).

Propensity matching was performed between each of the above three subgroups and the SHPT group. The matched factors were sex, diabetes, age (± 2 years), and dialysis vintage (± 12 months). After matching, low PTH Group 1 comprised 355 pairs; low PTH Group 2: 245 pairs, and low PTH Group 3: 202 pairs. The patients’ characteristics for each of the groups are shown in **Table 2**.

**Table 2:** Characteristics of the patients in the matched subgroups.

Characteristic	Group 1 (n = 355)	SHPT group (n = 355)	P value	Group2 (n = 245)	SHPT group (n = 245)	P value	Group 3 (n = 202)	SHPT group (n = 202)	P value
Median age (IQR)–year	57.3 (49.8–65.2)	57.7 (50.0–65.3)	0.559	58.2 (51.4–66.2)	58.7 (51.3–66.4)	0.201	56.5 (48.2–64.1)	57.0 (47.8–64.2)	0.141
Male sex – no. (%)	178 (50.1)	178 (50.1)	NA	116 (47.3)	116 (47.3)	NA	100 (49.5)	100 (49.5)	NA
Median dialysis vintage (IQR) –month	32.7 (7.6–62.9)	35.2 (12.2–65.2)	<0.001	33.7 (7.9–68.4)	37.5 (12.0–68.7)	0.010	60.7 (19.7–97.3)	60.3 (21.6–100.2)	0.521
Diabetes – no. (%)	112 (31.5)	112 (31.5)	NA	65 (26.5)	65 (26.5)	NA	52 (25.7)	52 (25.7)	NA
iPTH (pg/mL)			<0.001			<0.001			<0.001
Mean ± SD	80.6 ± 11.3	977.9 ± 458.8		45.1 ± 8.7	965.7 ± 437.1		15.8 ± 8.4	782.4 ± 278.3	
Median	80.7	812.5		45.1	783.7		16.3	691.8	
(IQR)	(72.1–90.0)	(683.3–1061.0)		(38.4–52.8)	(680.7–1061.3)		(8.7–22.9)	(633.5–799.8)	
Calcium (mmol/L)^*^	2.25 ± 0.21	2.28 ± 0.21	>0.900	2.28 ± 0.20	2.29 ± 0.23	0.737	2.26 ± 0.27	2.3 ± 0.23	0.142
Phosphate (mmol/L)^*^	1.64 ± 0.41	2.01 ± 0.41	<0.001	1.63 ± 0.40	1.97 ± 0.40	<0.001	1.62 ± 0.36	1.95 ± 0.39	<0.001
Albumin (g/dL)^*^	3.95 ± 0.30	3.83 ± 0.26	<0.001	3.90 ± 0.28	3.82 ± 0.26	0.010	3.93 ± 0.32	3.98 ± 0.24	0.339
Hemoglobin (g/dL)^*^	11.3 ± 0.8	11.2 ± 0.9	0.124	11.2 ± 0.8	11.2 ± 0.9	0.421	11.4 ± 0.9	11.4 ± 1.0	0.925

^*^Means ± SD.

Group 1, Low PTH Group 1 (60–100 pg/mL); Group 2, Low PTH Group 2 (30–60 pg/mL); Group 3, Low PTH Group 3 (<30 pg/mL).

iPTH, intact parathyroid hormone; IQR, interquartile range; NA, not applicable; SD, standard deviation; SHPT, secondary hyperparathyroidism.

### Low PTH Group 1 (iPTH 60–100 pg/mL) *Versus* the SHPT Group

**Figure 3:**
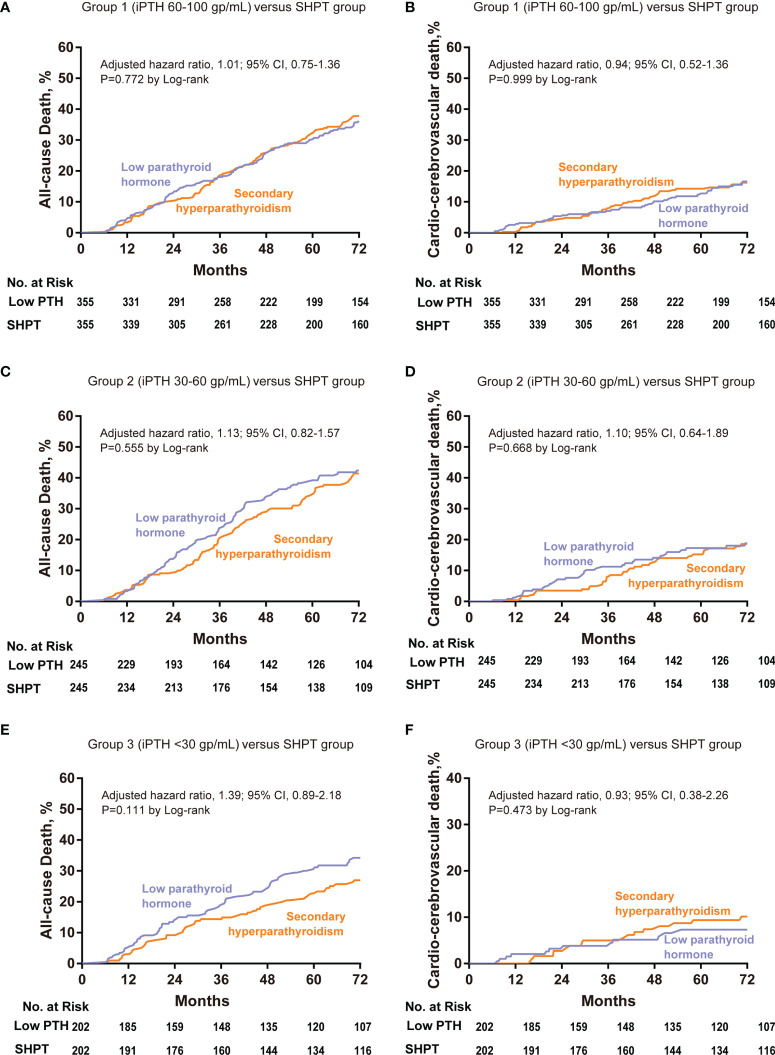
Cumulative event rates at 72 months for all-cause death and cardio-cerebrovascular death among patients with low PTH and SHPT in the propensity-matched cohorts. Low PTH group 1 (60–100 pg/mL) versus the SHPT group, all-cause death **(A)** and cardio-cerebrovascular death **(B)**. Low PTH group 2 (30–60 pg/mL) versus the SHPT group, all-cause death **(C)** and cardio-cerebrovascular death **(D)**. Low PTH group 2 (<30 pg/mL) versus the SHPT group, all-cause death **(E)** and cardio-cerebrovascular death **(F)**. Hazard ratios are based on multivariable Cox regression analyses. PTH, parathyroid hormone; SHPT, secondary hyperparathyroidism.

The cumulative incidences of all-cause death are depicted in **Figure 3A**. There was no significant difference between low PTH Group 1 and the SHPT group by log-rank testing (P=0.77). The results of the multivariable Cox regression analysis were consistent with this finding (P=0.96; HR=1.01; 95% CI: 0.75–1.36).

The cumulative incidences for cardio-cerebrovascular death are depicted in **Figure 3B**. We found no significant difference between low PTH Group 1 and the SHPT group by log-rank testing (P=0.99). The results of the multivariable Cox regression analysis were consistent with this finding (P=0.47; HR=0.94; 95% CI: 0.52–1.36).

### Low PTH Group 2 (iPTH 30–60 pg/mL) *Versus* the SHPT Group

The cumulative incidences of all-cause death are depicted in **Figure 3C**. We found no significant difference between low PTH Group 2 and the SHPT group by log-rank testing (P=0.56). The results of the multivariable Cox regression analysis were consistent with this finding (P=0.46; HR=1.13; 95% CI: 0.82–1.57).

The cumulative incidences of cardio-cerebrovascular death are depicted in **Figure 3D**. We found no significant difference between low PTH Group 2 and the SHPT group by log-rank testing (P=0.67). The results of the multivariable Cox regression analysis were consistent with this finding (P=0.72; HR=1.10; 95% CI: 0.64–1.89).

### Low PTH Group 3 (iPTH <30 pg/mL) *Versus* the SHPT Group

The cumulative incidences of all-cause death are depicted in **Figure 3E**. We found no significant difference between low PTH Group 3 and the SHPT group by log-rank testing (P=0.11). The results of the multivariable Cox regression analysis were consistent with this finding (P=0.15; HR=1.39; 95% CI: 0.89–2.18).

The cumulative incidences of cardio-cerebrovascular death are depicted in **Figure 3F**. We found no significant difference between low PTH Group 3 and the SHPT group by log-rank testing (P=0.47). The results of the multivariable Cox regression analysis were consistent with this finding (P=0.87; HR=0.93; 95% CI: 0.38–2.26).

### Low PTH Group Versus Medium PTH Group *Versus* SHPT Group

We divided the patients into the following three subgroups according to iPTH concentration: low PTH group (iPTH < 100 pg/mL), medium PTH group (iPTH 100–600 pg/mL), and SHPT group (≥600 pg/mL). Propensity matching was performed between all three groups. The matched factors were sex, diabetes, age (± 2 years), and dialysis vintage (± 12 months). After matching, 1641 cases were matched successfully in the three groups, and each group comprised 547 patients. The patients’ characteristics in each of the three groups are shown in **Table 3**.

**Table 3:** Characteristics of the patients in three matched groups: low PTH, SHPT, and medium PTH groups.

Characteristic	Low PTH group (n = 547)	SHPT group (n = 547)	Medium PTH group (n = 547)	P value
Median age	56.4	56,6	56.1	<0.001
(IQR)–year	48.1–64.5	48.3–64.5	47.6–64.5	
Male sex – no. (%)	277 (50.6)	277 (50.6)	277 (50.6)	NA
Median dialysis vintage (IQR) –month	51.3	50.9	49.7	0.026
	17.0–86.1	21.2–86.1	16.1–86.8	
Diabetes – no. (%)	131 (23.9)	131 (23.9)	131 (23.9)	NA
iPTH (pg/mL)				<0.001
Mean ± SD	55.5 ± 28.5	997.7 ± 452.6	297.1 ± 131.3	
Median	58.5	822.8	273.5	
(IQR)	31.6–79.8	693.2–1154.0	186.0–389.7	
Calcium (mmol/L)^*^	2.26 ± 0.23	2.30 ± 0.23	2.24 ± 0.22	<0.001
Phosphate (mmol/L)^*^	1.66 ± 0.42	2.01 ± 0.41	1.77 ± 0.44	<0.001
Albumin (g/dL)^*^	3.92 ± 0.33	3.92 ± 0.31	3.90 ± 0.37	0.498
Hemoglobin (g/dL)^*^	11.14 ± 1.12	11.10 ± 1.15	11.09 ± 1.22	0.064

^*^ Means ± SD.

Low PTH group (iPTH <100 pg/mL); SHPT group (iPTH ≥600 pg/mL); Medium PTH group (iPTH 100–600 pg/mL).

iPTH, intact parathyroid hormone; IQR, interquartile range; NA, not applicable; SD, standard deviation; SHPT, secondary hyperparathyroidism.

**Figure 4:**
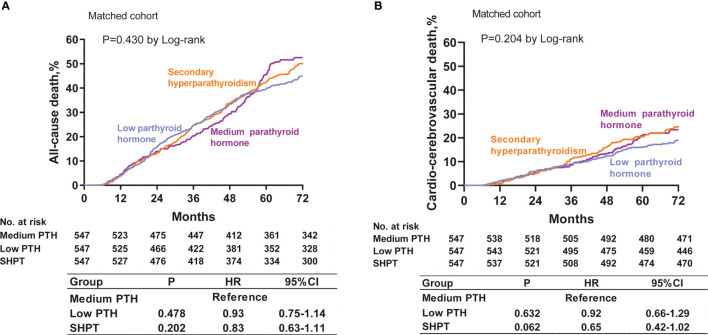
Cumulative event rates at 72 months for all-cause death **(A)** and cardio-cerebrovascular death **(B)** among patients with low PTH, medium PTH, and SHPT in the propensity-matched cohorts. Hazard ratios are based on multivariable Cox regression analyses. PTH, parathyroid hormone; SHPT, secondary hyperparathyroidism.

The cumulative incidences of all-cause death are depicted in **Figure 4A**. There was no significant difference between the low PTH group, medium PTH group, and SHPT group according to log-rank testing (P=0.430). The results of the multivariable Cox regression analysis were consistent with this finding.

The cumulative incidences for cardio-cerebrovascular death are depicted in **Figure 4B**. We found no significant difference between the low PTH group, medium PTH group, and SHPT group by log-rank testing (P=0.204). The results of the multivariable Cox regression analysis were consistent with this finding.

## Discussion

In this cohort study, we matched for age, sex, diabetes history, and duration of dialysis at baseline between the low PTH and SHPT groups to eliminate the cofounding effects of these known variables. We found that hemodialysis patients with low PTH (<100 pg/mL) and those with SHPT (>600 pg/mL) had comparable rates of long-term all-cause death and cardio-cerebrovascular death.

Some studies have shown that both low PTH and SHPT are associated with increased mortality in hemodialysis patients (2, 18). Low PTH may indicate adynamic bone disease, which weakens the buffering capacity of bone for fluctuations in serum phosphorus and calcium, which then may increase vascular calcification and mortality rates. Patients with SHPT have high serum PTH, which can promote the bone to release calcium and phosphorus into the serum. This leads to difficulty controlling serum calcium and phosphorus, which may increase mortality rates. However, these opinions are only speculations, based on the physiological effects of PTH. Evidence for a direct effect of PTH on vascular calcification and mortality in patients undergoing hemodialysis is lacking. Therefore, the association between serum PTH and mortality in hemodialysis patients is still controversial.

The results of the present study are consistent with those of some previous studies. A meta-analysis showed weak associations between serum PTH and the risk of death and cardiovascular events in patients with chronic kidney disease (7). However, this meta-analysis included all patients with chronic kidney disease, not only those undergoing hemodialysis. The association between PTH and mortality in hemodialysis patients has not yet been conclusively determined. In our previous study, subgroup analysis of age-matched groups found no statistically significant difference in mortality between patients with low PTH and those with SHPT (18). However, this subgroup analysis included only 36 patients and had a follow-up duration of 24 months. The present study was much larger and included propensity matching, which likely reduced the confounding effects of the matched variables. We concluded that the effect of PTH on the mortality rate of hemodialysis patients is much weaker than that of other strong, traditionally-recognized risk factors, such as age, diabetes history, and dry weight.

In our previous study (18), we found that hemodialysis patients with low PTH concentrations had a higher mortality rate than the rate in those with SHPT. Possible reasons for this discrepancy between our previous results and the results of the present study include the following: In the previous study, some very strong independent risk factors for mortality in hemodialysis patients were not balanced between the low PTH and SHPT groups, namely age (63.19 ± 10.63 versus 55.05 ± 14.09 years, respectively) and diabetes history (34.5% versus 24.5%, respectively). These imbalances may have impacted our findings. In the previous study, we performed multivariate analysis to minimize the confounding effects of the variables; however, the sample size was small, and there may have been residual confounding effects despite the multivariate analysis. In the present study, the low PTH group’s baseline characteristics were similar to those of the low PTH group in the previous study, namely older age and higher rate of diabetes compared with these variables in the SHPT group, and univariate analysis showed high mortality in the low PTH group. However, multivariate analysis identified no significant difference in mortality between the low PTH and SHPT groups after they had been matched.

Another study (Xi et al.) has shown that SHPT increases the risk of all-cause mortality, with patients with low PTH having the lowest mortality (19). Possible explanations for the discrepancy between these findings and our findings are as follows: First, the cohort in the study by Xi et al. comprised only hemodialysis patients who had undergone parathyroidectomy. Parathyroidectomy is generally performed when hemodialysis patients develop severe SHPT; such patients are more likely to benefit from parathyroidectomy, even if they subsequently have low PTH concentrations. Second, patients who can tolerate parathyroidectomy are generally in good general condition and have a longer life expectancy. Third, the patients in the study by Xi et al. were much younger than the patients in the present study (46.6 versus 59.4 years, respectively). These differences could account for patients with low PTH having the lowest mortality in the study by Xi et al.

Several limitations of this study need to be considered. First, this was an observational study; therefore, we could not identify any causal relationships. Second, some important risk factors, namely dry weight and blood pressure, were not matched in this study; we matched only for sex, age, diabetes, and dialysis vintage. Third, we were unable to access data on parathyroidectomy history or the treatment of metabolic bone disease, including with phosphorus binders, active vitamin D or its analogues, and calcimimetics. These treatments can affect serum concentrations of iPTH, which would affect the prognosis.

Our study was strengthened by the following two factors.: First, we performed propensity matching to balance many of the important risk factors for death, namely age, diabetes, dialysis vintage, and sex, between the low PTH and SHPT groups. Age and diabetes may be the two most important risk factors in hemodialysis patients and can therefore skew the findings, especially in small studies. Second, this was a large study with a relatively long follow-up period.

## Conclusions

Our findings suggest that hemodialysis patients with low PTH and those with SHPT have similar long-term outcomes in terms of all-cause death and cardio-cerebrovascular death. We found that even patients with very low PTH concentrations (< 30 pg/mL) had similar long-term outcomes to outcomes for those with SHPT. Further prospective studies are needed to confirm these findings.

## Data Availability Statement

The original contributions presented in the study are included in the article/supplementary materials, further inquiries can be directed to the corresponding author/s.

## Ethics Statement

The studies involving human participants were reviewed and approved by Bioethics Committee of Beijing Friendship Hospital, Capital Medical University. Written informed consent for participation was not required for this study in accordance with the national legislation and the institutional requirements.

## Author Contributions

WL and ZD conceived the study and contributed to the design of the research. WG and HZ performed the statistical analysis of the collected data. WG, YZ, and HH were involved in the preparation of the manuscript. All authors contributed to the article and approved the submitted version.

## Funding

This study was supported by the Beijing Municipal Administration of Hospitals Incubating Program (Code: PX2022003) and the Research Foundation of the Beijing Friendship Hospital, Capital Medical University (Code: YYQDKT2019-22).

## Conflict of Interest

The authors declare that the research was conducted in the absence of any commercial or financial relationships that could be construed as a potential conflict of interest.

## Publisher’s Note

All claims expressed in this article are solely those of the authors and do not necessarily represent those of their affiliated organizations, or those of the publisher, the editors and the reviewers. Any product that may be evaluated in this article, or claim that may be made by its manufacturer, is not guaranteed or endorsed by the publisher.
